# Percutaneous Large-Bore Pulmonary Thrombectomy with the FlowTriever Device: Initial Experience in Intermediate-High and High-Risk Patients

**DOI:** 10.1007/s00270-022-03266-0

**Published:** 2022-09-29

**Authors:** W. M. Luedemann, D. Zickler, J. Kruse, R. Koerner, J. Lenk, C. Erxleben, G. F. Torsello, U. Fehrenbach, M. Jonczyk, R. W. Guenther, M. De Bucourt, B. Gebauer

**Affiliations:** 1 Department of Diagnostic and Interventional Radiology, Charité Universitätsmedizin Berlin, Corporate member of Freie Universität Berlin and Humboldt-Universität Zu Berlin, Augustenburger Platz 1, 13353 Berlin, Germany; 2 Department of Nephrology and Medical Intensive Care, Charité Universitätsmedizin Berlin, Corporate member of Freie Universität Berlin and Humboldt-Universität Zu Berlin, Augustenburger Platz 1, 13353 Berlin, Germany

**Keywords:** Pulmonary embolism, Percutaneous embolectomy, Critical care, Extracorporeal membrane oxygenation

## Abstract

**Objectives:**

This retrospective cohort study investigates outcomes of patients with intermediate-high and high-risk pulmonary embolism (PE) who were treated with transfemoral mechanical thrombectomy (MT) using the large-bore Inari FlowTriever aspiration catheter system.

**Material and Methods:**

Twenty-seven patients (mean age 56.1 ± 15.3 years) treated with MT for PE between 04/2021 and 11/2021 were reviewed. Risk stratification was performed according to European Society of Cardiology (ESC) guidelines. Clinical and hemodynamic characteristics before and after the procedure were compared with the paired Student’s *t* test, and duration of hospital stay was analyzed with the Kaplan–Meier estimator. Procedure-related adverse advents were assessed.

**Results:**

Of 27 patients treated, 18 were classified as high risk. Mean right-to-left ventricular ratio on baseline CT was 1.7 ± 0.6. After MT, a statistically significant reduction in mean pulmonary artery pressures from 35.9 ± 9.6 to 26.1 ± 9.0 mmHg (*p* = 0.002) and heart rates from 109.4 ± 22.5 to 82.8 ± 13.8 beats per minute (*p* < 0.001) was achieved. Two patients died of prolonged cardiogenic shock. Three patients died of post-interventional complications of which a paradoxical embolism can be considered related to MT. One patient needed short cardiopulmonary resuscitation during the procedure due to clot displacement. Patients with PE as primary driver of clinical instability had a median intensive care unit (ICU) stay of 2 days (0.5–3.5 days). Patients who developed PE as a complication of an underlying medical condition spent 11 days (9.5–12.5 days) in the ICU.

**Conclusion:**

In this small study population of predominantly high-risk PE patients, large-bore MT without adjunctive thrombolysis was feasible with an acceptable procedure-related complication rate.

**Supplementary Information:**

The online version contains supplementary material available at 10.1007/s00270-022-03266-0.

## Introduction

Venous thromboembolism is the leading cause of preventable death among hospitalized patients, the third most common cause of cardiovascular death worldwide after stroke and myocardial infarction and strongly associated with age [1–4].

For intermediate-high-risk and high-risk pulmonary embolism (PE), the 2019 European Society of Cardiology (ESC) treatment guidelines recommend hospital admission, systemic anticoagulation and supportive care [5–7]. Systemic thrombolysis can be performed to reduce the risk of acute RV failure, all-cause mortality and recurrence rates of PE [8]. Given the associated risks of bleeding, it is reserved for unstable patients and does not improve the incidence of post-PE syndromes [9–12]. Surgical embolectomy is a potential alternative for selected patients but associated with significant mortality and morbidity [13]. Interventional treatment options for unstable patients include catheter-directed low-dose thrombolysis (CDT), thrombus fragmentation and mechanical thrombectomy (MT) [14–18]. Recent data cast doubt on the additional benefit of CDT in terms of thrombus removal and safety in high-risk PE patients [19–21]. Thrombus fragmentation carries the risk of distal embolization and was demonstrated inferior to aspiration with dedicated catheters in the treatment of large-burden PE in a recent in vitro study [20]. That shifts the focus on new devices designed for pulmonary thrombectomy. Dedicated devices currently used for MT in the pulmonary vasculature are the 8F Penumbra Indigo aspiration system (Penumbra Inc, Alameda, California) and the large-bore 24F Inari FlowTriever system (Inari Medical, Irvine, California), both with encouraging preliminary safety and efficacy data, particularly in intermediate-risk PE [22–25].

The Inari FlowTriever is a single-use MT system and received U.S. Food and Drug Administration 510(k) clearance for PE in 2018 and the conformité européenne mark in late 2020. The FlowTriever Pulmonary Embolectomy Clinical Study (FLARE), a prospective, single-arm, multicenter investigational device exemption trial, enrolled 106 patients with intermediate-risk PE. A mean reduction of the right-to-left ventricular (RV/LV) ratio of 25.1% was achieved at a major adverse event rate of 3.8% and with a mean intensive care unit (ICU) stay of 1.5 days [23]. A retrospective single-center cohort study of 46 patients with intermediate and high-risk PE echoed these findings [24]. A propensity score analysis published in 2021 comparing MT with the FlowTriever and routine care found MT to decrease in-hospital mortality and decrease ICU length of stay for patients with intermediate or high-risk PE [25].

The purpose of the study is to report on our initial experience and safety outcomes with the Inari FlowTriever system for MT in a predominantly high-risk PE collective.

## Materials and Methods

### Patient Enrollment and Study Design

This retrospective single-institution cohort study was approved by the institutional review board (EA4/021/22). Patients with intermediate-high-risk or high-risk acute PE who failed to improve after thrombolysis or those with contraindications to thrombolysis were considered eligible for MT following current recommendations [5]. Between April 2021 and November 2021, all patients with follow-up data until the end of their hospital stay were included for retrospective analysis. There were no exclusion criteria.

### Definitions and Outcome Parameters

The type and frequency of adverse advents that may or not may be procedure-related were assessed and classified according to the Cardiovascular and Interventional Radiological Society classification system of complications [26].

Before and after MT, the heart rate and invasive pulmonary artery pressures (PAP) were measured whenever feasible. As no clinical decision was based on a specific pressure measurement, these could be omitted in high-risk patients for the sake of time.

The Simplified Acute Physiology Score (SAPS) II is a routinely calculated mortality estimation tool based on the worst values of 12 physiological variables and 3 disease-related variables within the first 24 h of ICU admission. The score was used to characterize the clinical impairment of patients before and after MT. With a SAPS II score between 30 and 40 points, the mortality risk ranges from 7.9 to 15%, and with a score between 40 and 50, the risk is expected in the range of 15–26.6% [27–29]. In addition, pre- and post-interventional circulatory and respiratory support requirements were assessed.

The cohort was divided into patients who were either admitted for PE or who were previously hospitalized and developed PE as a complication. The respective post-interventional clinical courses were compared.

### Data Collection

Retrospective data collection was performed using our radiological information system and picture archiving and communication system as well as our ICU patient data management system (COPRA System, Berlin, Germany).

### Interventional Thrombectomy Procedure

Board-certified interventional radiologists (15, 18, 7 years of experience) and advanced interventional radiology fellows (2 and 3 years of experience) performed the interventions. The FlowTriever system is a single-use 16F, 20F or 24F aspiration catheter. It is operated through a 24F Gore DrySeal sheath (Gore, Flagstaff, Arizona, USA) and directed into the PA over a 0.035’’ superstiff Amplatz wire (Boston scientific, Marlborough, Massachusetts, USA). A vacuum is created in a 60-ml syringe, and opening of a stopcock enables thrombus aspiration. For more adherent thrombi, a triplet of self-expanding mesh disks in different sizes mounted on a dedicated catheter can be deployed for mechanical thrombus disruption. Before and after MT, thrombus burden was assessed with DSA and pulmonary artery pressures (PAP) were taken whenever feasible. DSA was repeated at the discretion of the interventional radiologist, and a final DSA was performed before device removal (Supplement 1, Supplement Figs. 1 and 2 ). The primary endpoint was visual thrombus removal on DSA. Residual thrombus was accepted if patients showed clinical improvement in terms of reduced oxygen or vasopressor requirements. Anticoagulation preferably with unfractionated heparin with a target activated clotting time of 240 s is recommended in order to prevent clotting of the aspiration catheter.

### Statistical Analysis

All statistical analyses were conducted with IBM SPSS STATISTICS, version 25 (IBM Corporation, Armonk, NY, USA). Normally distributed continuous data are presented with mean and standard deviation or number and percentage. Non-normally distributed data are expressed as median with the 95% confidence interval (95% CI). Comparisons of clinical and hemodynamic characteristics before and after the index procedure were conducted with paired Student’s *t* tests. Duration of hospital stay was analyzed with Kaplan–Meier curves and compared with the log-rank test; patients that were transferred to other facilities were censored. A *p* value < 0.05 was considered to indicate statistical significance.

## Results

### Baseline Demographics

**Fig. 1:**
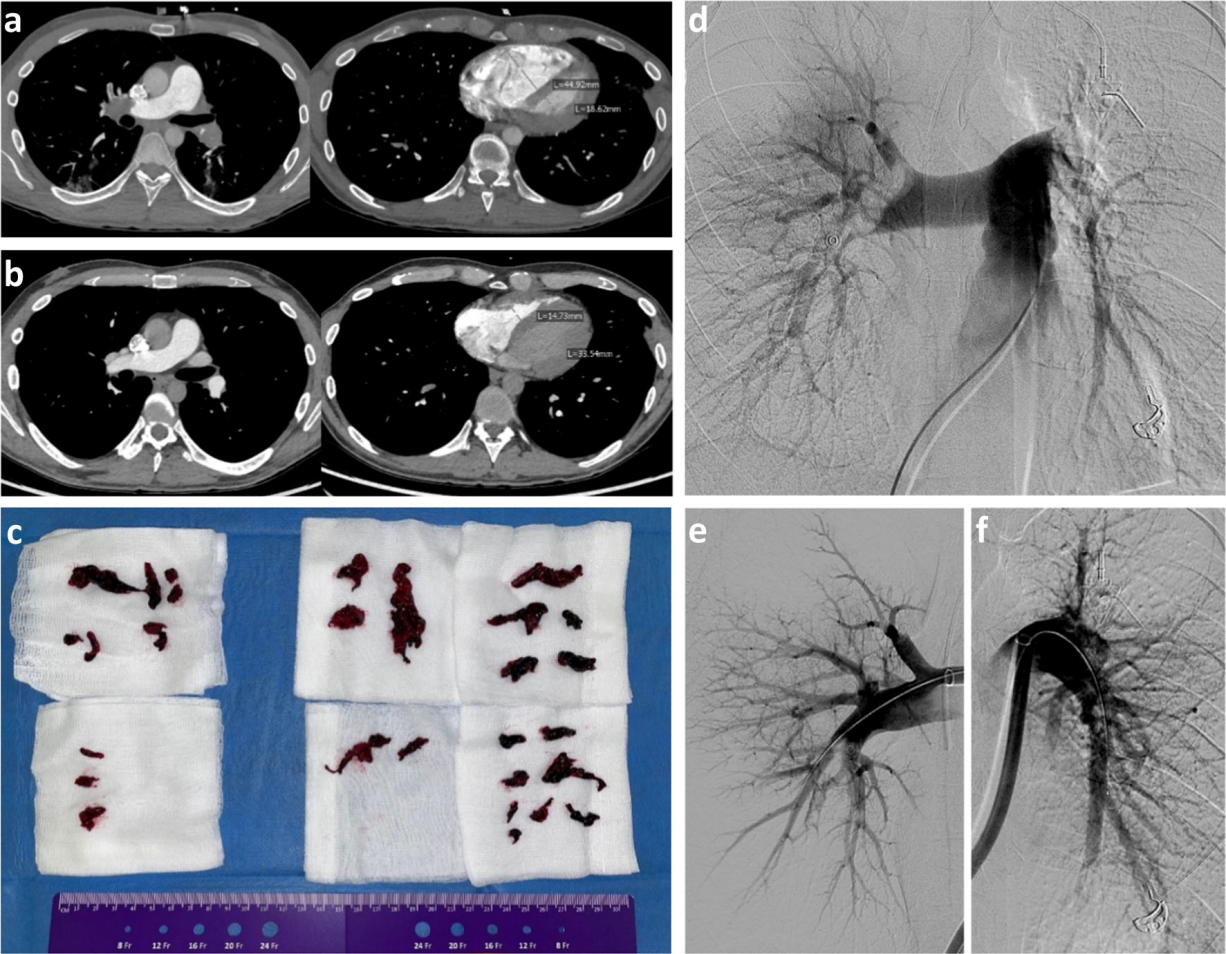
CT and angiograms from a representative thrombectomy case. Images of a 31-year-old male patient undergoing chemotherapy for colorectal carcinoma. Contrast-enhanced CT at the level of the pulmonary arteries (left) and at the level of the ventricles (right) before (**a**) and 1 week after thrombectomy (**b**) with the 24F device. The right ventricle is decompressed after successful thrombectomy. Right-to-left ventricular ratio improved from 2.4 to 0.43. Digital subtraction angiograms before (**d**) and after (**e**, **f**) thrombectomy. Extracted clots are shown in (**c**). The mean pulmonary artery pressure dropped from 35 to 16 mmHg, and the heart rate decreased from 130 to 85 beats per minute. Intensive care unit duration of stay was 2 days, hospital stay was 7 days

Between April 2021 and November 2021, 27 patients were included for retrospective analysis (Table 1). The mean patient age was 56.1 years, (± 15.3 years) and 15/27 were female. 18/27 patients were classified as high-risk PE according to ESC criteria, and nine patients were classified as intermediate-high-risk (Table 2). A representative case is depicted in Fig. 1.

**Table 1:** Baseline demographic data

Age (years)	56.1 ± 15.3
Female	15 (55.6)
BMI, kg/m^2^	29.6 ± 7.1
COPD	1 (3.7)
Coronary artery disease	1 (3.7)
Arterial hypertension	10 (37)
Prior or subacute stroke	4 (14.8)
Chronic renal insufficiency	1 (3.7)
Tumor	7 (25.9)
*Cause of hospital admission*	
Pulmonary embolism	18 (66.7)
COVID-19	4 (14.8)
Other	5 (18.5)
*Contraindications for thrombolysis*	
Active bleeding or recent surgery	5 (18.5)
Recent intracranial bleeding	2 (7.4)
Subacute stroke	3 (11.1)
Head trauma	2 (7.4)
ECMO	4 (14.8)
COVID-19	2 (7.4)
Other (relative contraindications)	7 (25.9)
Prior thrombolysis not effective	2 (7.4)
Concomitant DVT proven	13 (48.1)

Values are *n* (%) or mean ± SD

*BMI* body mass index, *COPD* chronic obstructive pulmonary disease, *COVID-19* coronavirus disease 2019, *DVT* deep vein thrombosis, *ED* emergency department, *ECMO* extracorporeal membrane oxygenation

**Table 2:** Pulmonary embolism severity

Acute clinical impairment	
*sPESI score*	
0	0 (0)
1	27 (100)
*ESC class*	
Intermediate-high-risk	9 (33.3)
High-risk	18 (66.7)
Pre-interventional SAPS II score	36.8 ± 14.8
Heart rate in beats per minute	109.4 ± 22.5
Pre-interventional vasopressor support	18 (66.7)
Patient on 1 vasopressor	7 (25.9)
Patient on > 1 vasopressor	11 (40.7)
*Biomarker elevation (available data)*	
D-dimer (20/27)	20 (100)
Cardiac troponin T (21/27)	18 (85.7)
Brain natriuretic peptide (20/27)	16 (80)
Lactate (27/27)	18 (66.7)
Patient intubated	16 (80)
ECMO	5 (18.5)
VA-ECMO	4 (14.8)
VV-ECMO	1 (3.7)
*Imaging findings on pulmonary CTA*	
*Pulmonary embolism location*	
Unilateral right	1 (3.7)
Unilateral left	1 (3.7)
Bilateral and central	25 (92.6)
Right-to-left ventricular ratio	1.7 ± 0.6
Contrast reflux into the IVC	20 (74.1)
Diameter of the pulmonary trunk	31.8 ± 4.5
Pulmonary infarct	13 (48.1)
*Imaging findings on ECHO (available data)*	
TAPSE in mm (13/27)	19.5 ± 8.6
sPAP in mmHg (11/27)	54.1 ± 21.5

Values are *n* (%) or mean ± SD

*ECMO* extracorporeal membrane oxygenation, *ESC* European Society of Cardiology, *IVC* inferior vena cava, *SAPS II score* simplified acute physiology score II, *sPAP* systolic pulmonary artery pressure, *sPESI* simplified pulmonary embolism severity score, *TAPSE* tricuspid annular plane systolic excursion, *VA* veno-arterial, *VV* veno-venous

### Procedural Characteristics

All patients were started on systemic anticoagulation before the intervention, in most cases with unfractionated heparin (25/27). In all patients, a right femoral access was used. MT was performed with a combination of the 24F and the 16F device in 12/27 cases. In 10/27 cases, the 24F device only was used. Additional disk catheters were used in 4 cases. Mean procedure time was 134.6 min (Table 3). Technical success without clinical improvement was seen in two patients with prolonged cardiogenic shock who later succumbed to brain edema (Table 3).

**Table 3:** Procedural characteristics and patient safety

Procedure time in minutes	134.6 ± 50.4
*Devices used*	
T24 only	10 (37.0)
T24 and T16	12 (44.4)
Any combination of T24, T20 or T16	5 (18.5)
Disks	4 (14.8)
*Anticoagulation*	
UFH	25 (92.6)
LMWH	1 (3.7)
Argatroban	1 (3.7)
*Adverse events (CIRSE complication grade)*	
Peri-interventional CPR (grade 1)	2 (7.4)
Post-interventional blood transfusions (grade 3)	5 (19)
Prior surgery/ intraabdominal bleeding	3
Bleeding from ECMO cannulation site	2
Death (grade 6)	5 (19)
Stroke due to paradoxical embolism (PFO) before MT	1
Stroke due to paradoxical embolism (PFO) after MT	1
In-house STEMI > 7 days after MT	1
Prolonged cardiogenic shock and brain edema	2

Values on the right are *n* (%) or mean ± SD

*CIRSE* Cardiovascular and Interventional Radiological Society, *UFH* unfractionated heparin, *LMWH* low-molecular-weight heparin, *CPR* cardiopulmonary resuscitation, *ECMO* extracorporeal membrane oxygenation, *PFO* persistent foramen ovale, *STEMI* ST elevation myocardial infarction

### Safety Outcomes

Cardiopulmonary resuscitation (CPR) was necessary in 2/27 patients. In one patient, this was due to contralateral displacement of a clot that could initially not be aspirated with the T24 device. The patient stabilized after successful aspiration of the dislocated thrombus. Another patient deteriorated right after the femoral access was established and required intubation and short CPR. One patient with COVID-19 pneumonia was intubated before the procedure and required intensified respiratory support after the intervention. Post-interventional blood transfusions were necessary in 5/27 patients who had additional blood losses from surgery (3/27) or bleeding at ECMO cannulation sites (2/27). No major bleeding complications from the puncture sites for the device were reported. All-cause mortality was 5/27 patients. Two patients did not recover from prolonged cardiogenic shock and succumbed to brain edema. One patient was diagnosed with stroke in the posterior circulation and PFO 1 week after the procedure and died of hemorrhagic transformation. In one patient, stroke due to paradoxical embolism, which later proved to be fatal, was the contraindication to thrombolysis and the reason why MT was performed. One patient died of ST elevation infarction 11 days after MT. There was no need for additional thrombolysis in any case. Adverse events are summarized and classified in Table 3.

### Peri-interventional effectiveness outcomes and post-interventional clinical course.

**Fig. 2:**
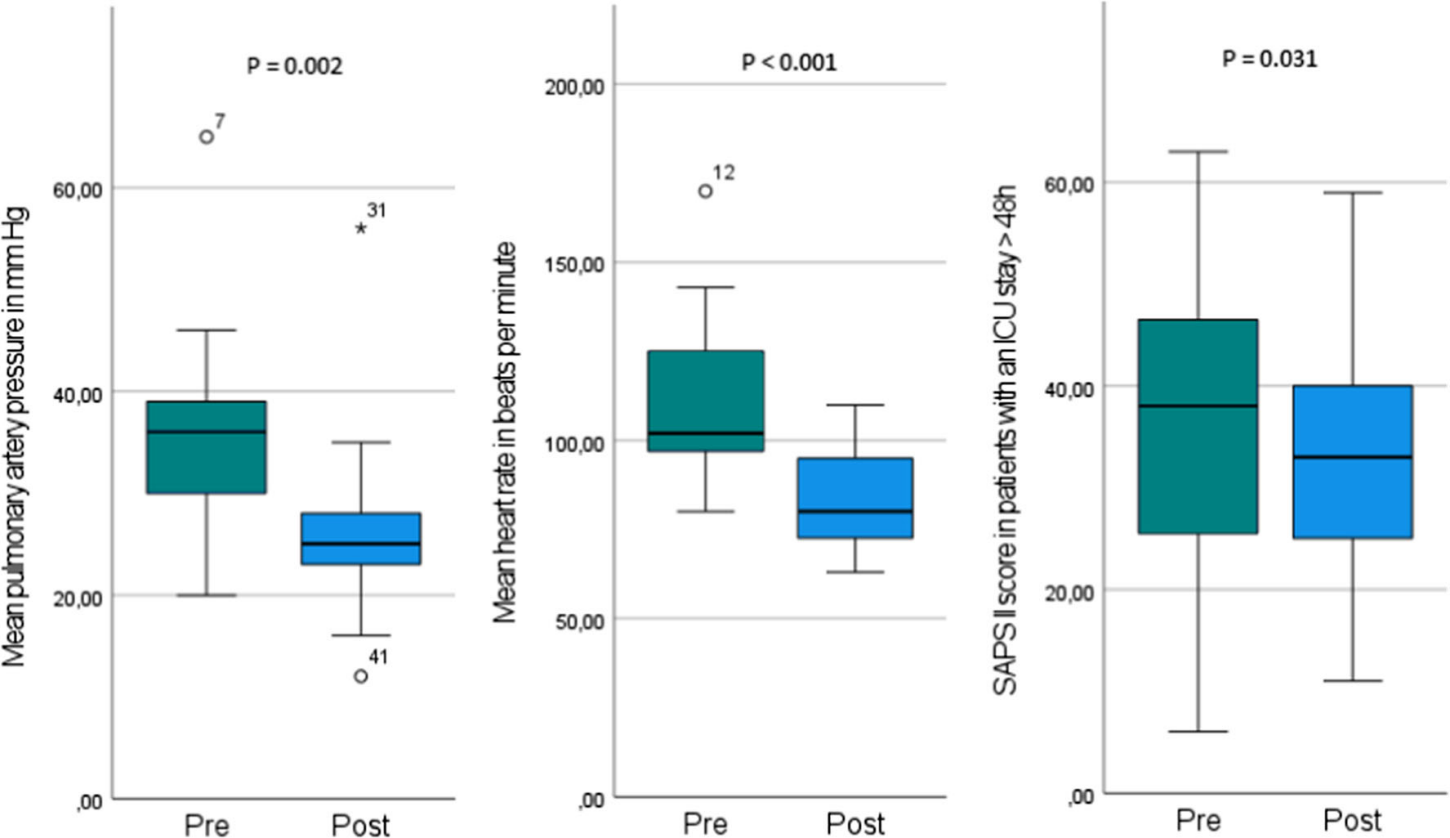
Peri-procedural outcome measures. Mean pre-interventional pulmonary artery pressure (PAP) was 35.9 ± 9.6 mm Hg and decreased to 26.1 ± 9.0 mm Hg on average. Mean heart rate dropped from 109.4 ± 22.5 beats per minute (bpm) to 82.8 ± 13.8 bpm. The simplified Acute Physiology Score (SAPS) II is a disease severity score and mortality estimation tool that integrates the worst values of 12 physiological variables and 3 disease-related variables within the first 24 h of ICU admission. A second score was calculated if patients spent at least 48 h in the ICU. In 23/27 patients, two scores were available which decreased from mean 37.8 ± 15.3 points to an average of 31.2 ± 13.3 points

Invasive pre- and post-interventional invasive PAP measurements were available in 20/27 patients, and the heart rate was documented in all patients. Data on the SAPS II score before and after the intervention were available in 23/27 patients. Patients who either deceased (1/27) or could be discharged from the ICU (3/27) within the first 48 h after admission did not have a second SAPS II score calculated. A significant decrease was seen for mean PAP (*p* = 0.002), heart rate (*p* < 0.001) and SAPS II scores (*p* = 0.02) (Fig. 2, Supplement Table 1). In 16/27 patients, vasopressor requirements decreased or veno-arterial ECMO blood-flow was reduced within 24 h after the procedure. In 20/27 patients, the ventilation support could be decreased (Table 4). Median ICU stay for patients who were admitted to the hospital for acute PE was 2 days compared to 11 days when PE occurred in previously hospitalized patients. Median hospital stay was 7 and 20 days, respectively.

**Table 4:** Post-interventional clinical course

Reduction of circulatory support within 24 h	16 (88.9)
Reduction of ventilation support within 24 h	20 (74.1)
Median ICU stay in days (95% CI) *	5 (1.9–8.1)
PE only	2 (0.5–3.5)
PE as complication	11 (9.5–12.5)
Median hospital stay in days (95% CI) *	14 (7.8–20.2)
PE only	7 (0.8–13.2)
PE as complication	20 (12.1–27.9)
*Discharge*	
Home	16 (59.3)
Rehabilitation facility/ other hospital	6 (22.2)
Death	5 (18.5)

Values are *n* (%) or mean ± SD

*CI* confidence interval, *ICU* intensive care unit, *PE* pulmonary embolism

*Duration of hospital stay was analyzed with Kaplan–Meier curves and compared with the log-rank test; patients that were transferred to other facilities were censored

## Discussion

This retrospective, single-center cohort study investigated outcomes of patients treated for PE with the Inari FlowTriever large-bore aspiration catheter system. Most patients were classified as high-risk PE. We report one device-related serious adverse event which was contralateral thrombus dislocation treated successfully with thrombus extraction. In another patient with PFO and fatal stroke due to paradoxical embolism, MT could have caused the event. Blood transfusions after MT were necessary in previously anemic patients, whereas blood losses after MT alone did not require transfusion. Invasive measurements showed a significant reduction in PAP and heart rate following MT, followed by reduced needs for circulatory and respiratory support as well as lower aggregate scores of clinical impairment the day after. When PE was the primary driver of clinical instability, we saw prompt patient recovery with a median ICU stay of 2 days.

As underlined by the relevant rate of adverse events, high-risk PE patients are critically ill and may perform poorly irrespective of MT. Critical deterioration directly related to the intervention, however, is uncommon and could be preempted, e.g., through ruling out of a PFO with echocardiography or exerting extreme caution with clots that are difficult to aspirate despite the recommended waiting time under vacuum of at least 5 min. To minimize the risk of clot dislocation, we advocate for thorough use of the included nitinol disks to break down the clot as much as possible before aspiration. Blood loss and transfusion requirements are device-related in risk collectives and have been reported previously [24]. In the near future, a dedicated blood return system will most likely minimize this issue. Unfortunately, it is not yet CE-certified. We attribute the observed clinical improvement after MT to the immediate reduction of right ventricular afterload, although causality cannot be inferred from our observational data.

Our results are consistent with data from the FLARE Study in which patients with acute intermediate-risk PE were treated with the device and we do not see higher rates of intervention- or device-related clinical deterioration [23]. With respect to recently published safety outcomes for CDT in a high-risk PE cohort that detailed major bleedings in 12/33 patients, MT seems to entail less procedure-related adverse events in high-risk PE patients [20]. One retrospective study employed propensity score matching of patients who either received routine care or MT with the FlowTriever device. The authors reported an in-hospital mortality of patients treated with conservative care of 23.3% and significantly lower rates in the MT group of 3.6% [25]. This is lower than the 7.4% (2/27 patients) we report to have directly succumbed to PE, although our patients died of brain edema due to prolonged cardiogenic shock prior to MT. In agreement with our data, the authors reported a median ICU length of stay of 2 ± 1.2 days in patients who were hospitalized for acute PE. This was significantly lower than the stay of patients who received routine care only, which was reported as 6.1 ± 8.6 days. The reduction in PAP we observed was greater than previously reported, which is probably owed to our high percentage of high-risk patients with acutely elevated PAP [23, 24].

Our findings are clinically relevant in several ways. Many critically ill patients, e.g., with COVID-19 pneumonia are anticoagulated and have relative contraindications to thrombolysis [9, 19, 30]. Our safety data are meant to reduce potential reservations for MT with the FlowTriever device in high-risk PE patients. We provide encouraging observational data on the efficacy of the device and echo prior findings that MT of PE can potentially shorten ICU stays in comparison to conservative treatment or systemic thrombolysis which may be beneficial in preserving everyday functionality in elderly patients. It remains to be shown in further studies, how the system holds up against the 8F Penumbra Indigo Aspiration System which likewise showed a significant reduction in the RV/LV ratio and a low major adverse event rate in intermediate-risk PE patients in a prospective, multicenter study published in 2021 [22].

There are a number of limitations to the generalizability of our results. The retrospective study design makes it difficult to assess the magnitude of our treatment effect. It would have been insightful to provide a propensity score-matched cohort that received thrombolysis as an active comparator. However, as the majority of patients had absolute contraindications to lysis, this was not feasible. Furthermore, data collection was not complete in all cases and especially transthoracic echocardiography (TTE) data were fragmentary. This is a pity as right-ventricular strain is most frequently assessed and followed-up with bedside TTE. It is the imaging modality referring physicians are most comfortable with and we would have liked to provide more insightful data on its role in early follow-up of MT.

## Conclusion

Large-bore MT of PE with the Inari FlowTriever device can be performed with an acceptable safety-profile even in high-risk PE patients. Device-related and procedure-related complications should be considered in a risk–benefit profile with respect to the gold standards of anticoagulation and thrombolysis. Observational data on its efficacy are encouraging, but randomized-controlled trials are warranted and also should investigate the incidence of post-PE syndromes.

## Supplementary Information

Below is the link to the electronic supplementary material.

Supplementary file1 (DOCX 5666 KB)

## Abbreviations

CDT: Catheter-directed thrombolysis
CE: Contrast enhanced
CI: Confidence interval
COVID-19: Coronavirus disease 2019
CPR: Cardiopulmonary resuscitation
ECMO: Extracorporeal membrane oxygenation
ESC: European Society of Cardiology
ICU: Intensive care unit
IQR: Interquartile range
MT: Mechanical thrombectomy
OS: Overall survival
PA: Pulmonary artery
PAP: Pulmonary artery pressure
PE: Pulmonary embolism
RV: Right-ventricular
SAE: Serious adverse event
SAPS II: Simplified Acute Physiology Score (SAPS) II
sPAP: Systolic pulmonary artery pressure (as measured with echocardiography)
TAPSE: Tricuspid annular plane systolic excursion
TTE: Transthoracic echocardiography
