# Supplementary material for: Percutaneous Large-Bore Pulmonary Thrombectomy with the FlowTriever Device: Initial Experience in Intermediate-High and High-Risk Patients
Source: Cardiovasc Intervent Radiol. 2022 Sep 29;46(1):35–42. doi: 10.1007/s00270-022-03266-0 (PMC9521880; doi:10.1007/s00270-022-03266-0)
Supplement: Supplementary file 1 — Supplementary file1 (DOCX 5666 KB) [file 270_2022_3266_MOESM1_ESM.docx]

**Supplements**

**Supplement 1:** Interventional thrombectomy protocol

Board-certified interventional radiologists and advanced interventional radiology fellows performed the interventions according to a standard protocol. All patients had a prior contrast-enhanced CT scan to confirm the diagnosis of at least sub-massive pulmonary embolism. Anticoagulation with unfractionated heparin is the standard of care at our institution and recommended by the manufacturer to prevent thrombosis of the FlowTriever aspiration catheter during intervention.

Ultrasound of the groin was performed to assess for thrombus burden in the access vessel. After skin disinfection, sterile draping and local anesthesia, usually the right common femoral vein was accessed under ultrasound guidance and a 5F sheath (Cordis, Miami Lakes, Florida, USA) was inserted in Seldinger technique. An initial phlebography was performed to assess relevant thrombus burden in the femoral, iliac and caval veins. A 5F catheter in pigtail configuration (Cordis, Miami Lakes, Florida, USA) was advanced into the right jugular vein which allowed for the safe placement of a 0.035’’ Amplatz superstiff wire with a 1 cm short, straight tip (Boston scientific, Marlborough, Massachusetts, USA). Dilatations with dilatators in increasing size over the Amplatz wire facilitated the insertion of a 24F Gore DrySeal sheath (Gore, Flagstaff, Arizona, USA) with tip position within the inferior vena cava. The tricuspid valve was passed with a pigtail catheter which was then advanced to the level of the pulmonary trunk with mid-catheter? support through a manually curved 0.035’’ Bentson wire (Cook medical, Bloomington, Indiana, USA) (see figure 1 below). A digital subtraction angiography (DSA) was performed to assess the thrombus burden and pulmonary artery pressure (PAP) measurements were taken. The Bentson wire and the pigtail catheter were exchanged for a 0.035’’ hydrophilic guide wire (Terumo Europe) and a 5F or 4F vertebralis catheter. In bilateral thrombus burden, the right pulmonary artery was usually treated first and the vertebralis catheter was placed in the lower lobe artery as distally as possible in order to safely exchange the hydrophilic guide wire for an 0.035” Amplatz superstiff wire (1 cm floppy tip length). Now the 24F or 20F FlowTriever aspiration guide catheter was advanced to the level of the right or left pulmonary artery, just proximal to the occlusive thrombus. The plunger of the large-bore 60 ml syringe was pulled back to create a vacuum yet withheld by the closed two way stop-cock. The vacuum was then released by opening the two way stop-cock, thus abruptly generating an aspirational force of up to 150 ml/s while also limiting blood loss to 60 ml. Additional aspirations in different positions were made until there was no relevant residual thrombus burden. For more wall-adherent thrombus, a triplet of self-expanding mesh disks mounted on a dedicated catheter were deployed through the aspiration catheter. The disks are available in different sizes (6 to 10, 11 to 14, and 15 to 18 mm) to match vessel diameters in order to engage, fragment/ disrupt and pull the thrombus toward the aspiration catheter in stent retriever fashion. Segmental thrombus burden was accessed with a 16F aspiration catheter through the 20F or 24F outer catheter. After completion of thrombectomy, another PAP measurement and pulmonary angiography was performed and the devices were removed. The puncture site was adapted with a z-stitch or purse-string suture and/or compressed manually.

**Supplement figure 1** Navigating the pigtail catheter past the tricuspid valve


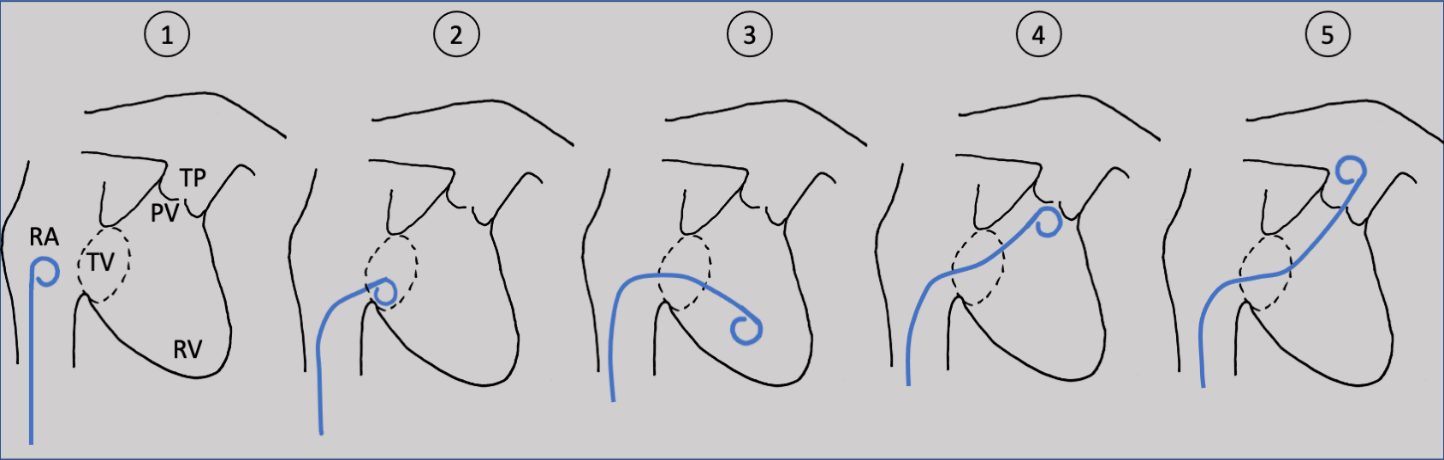

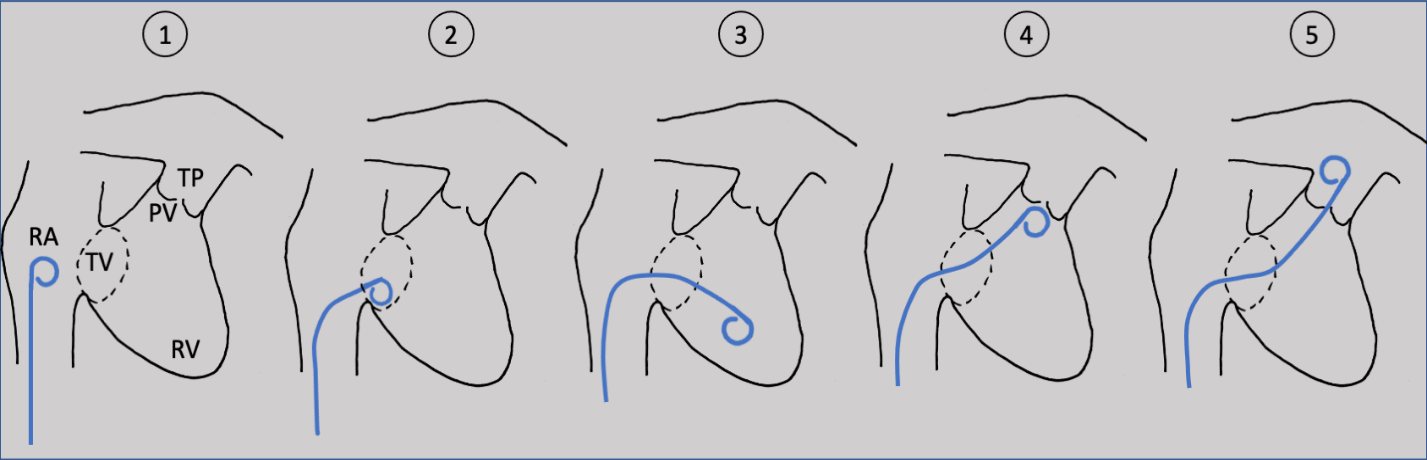


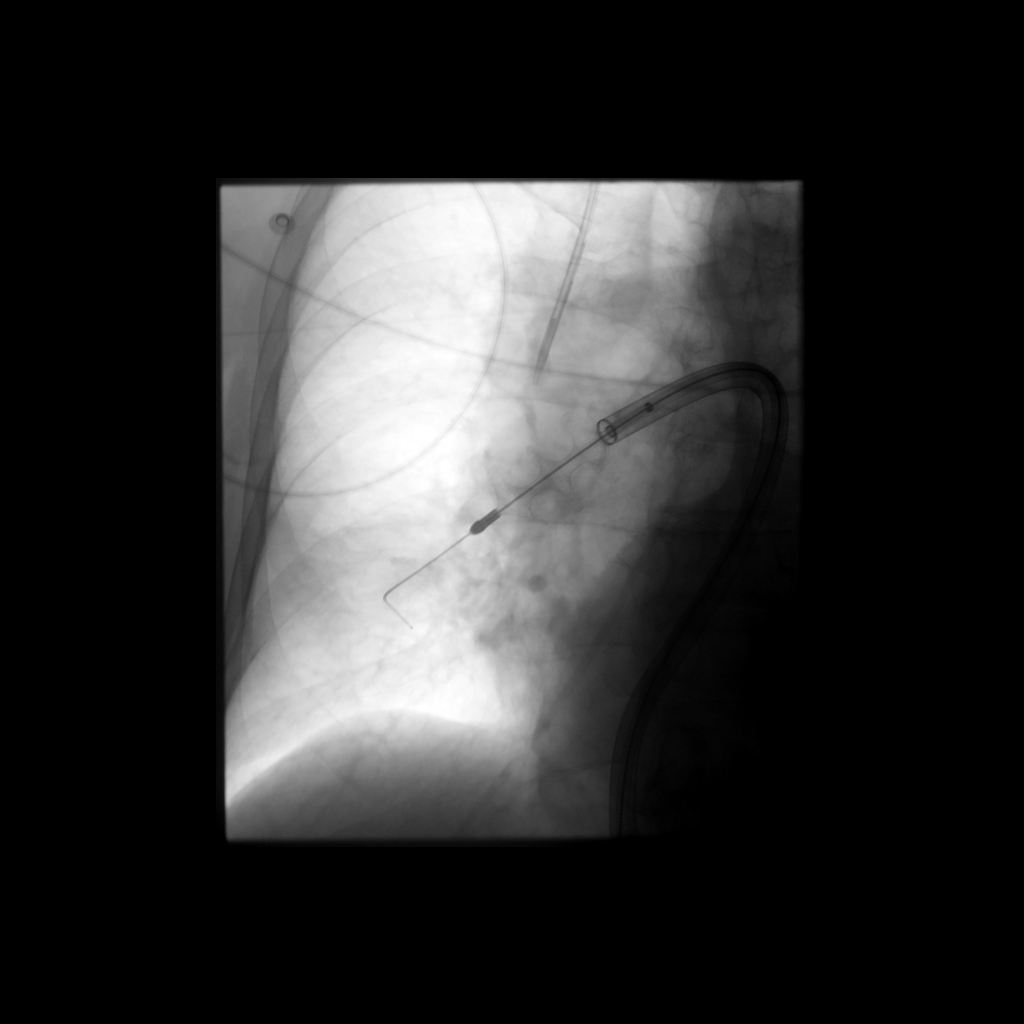


**g**

**h**


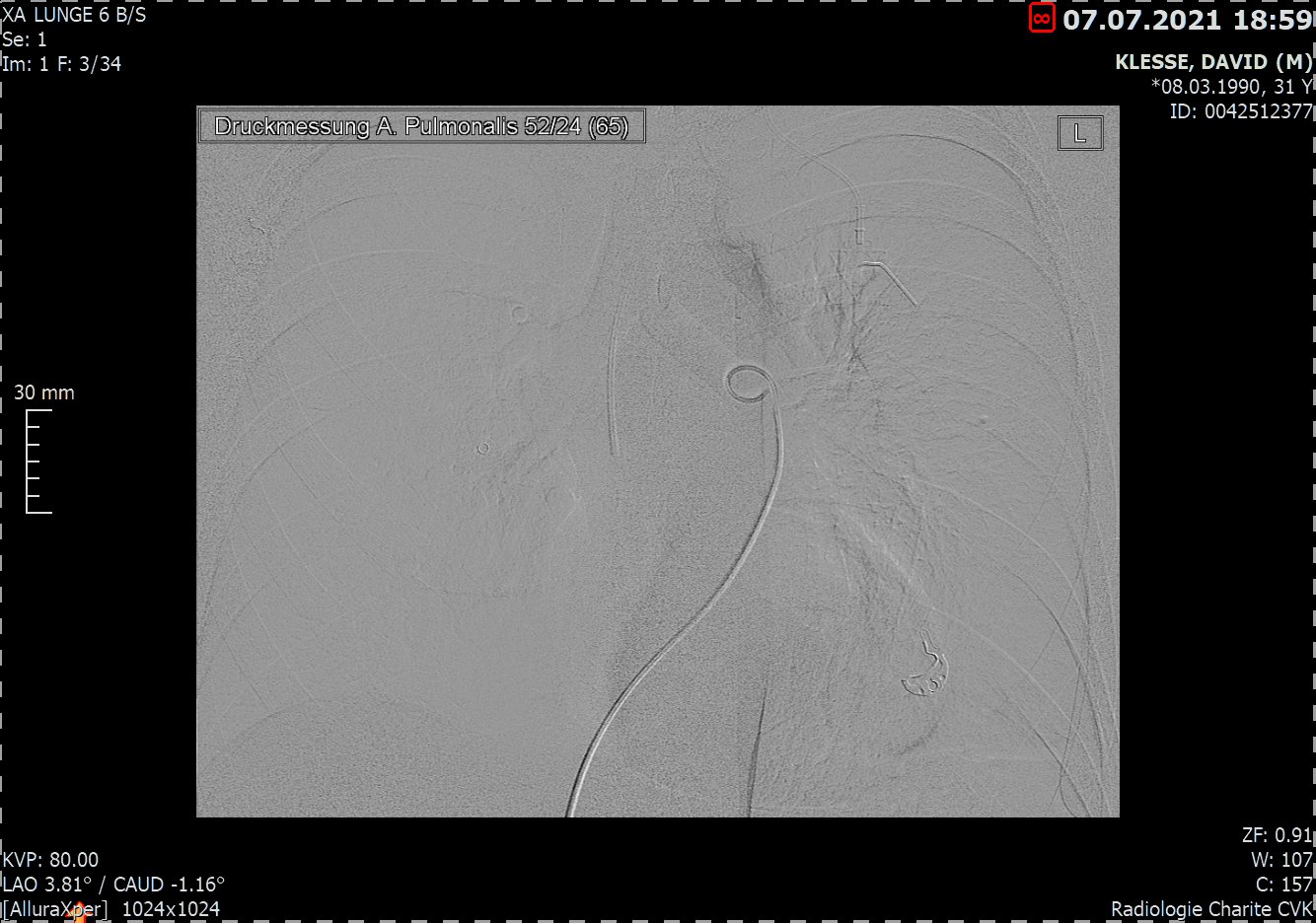

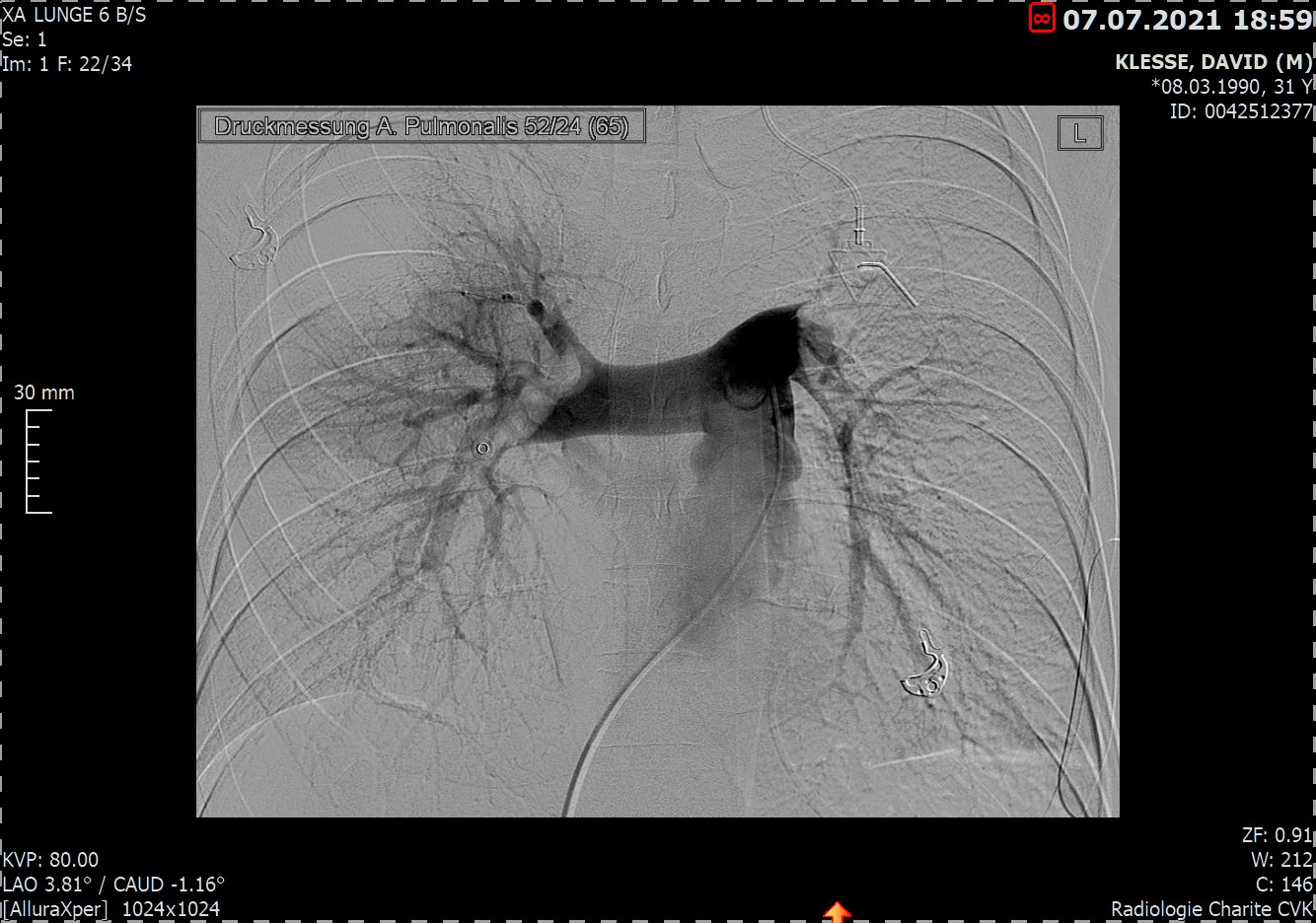

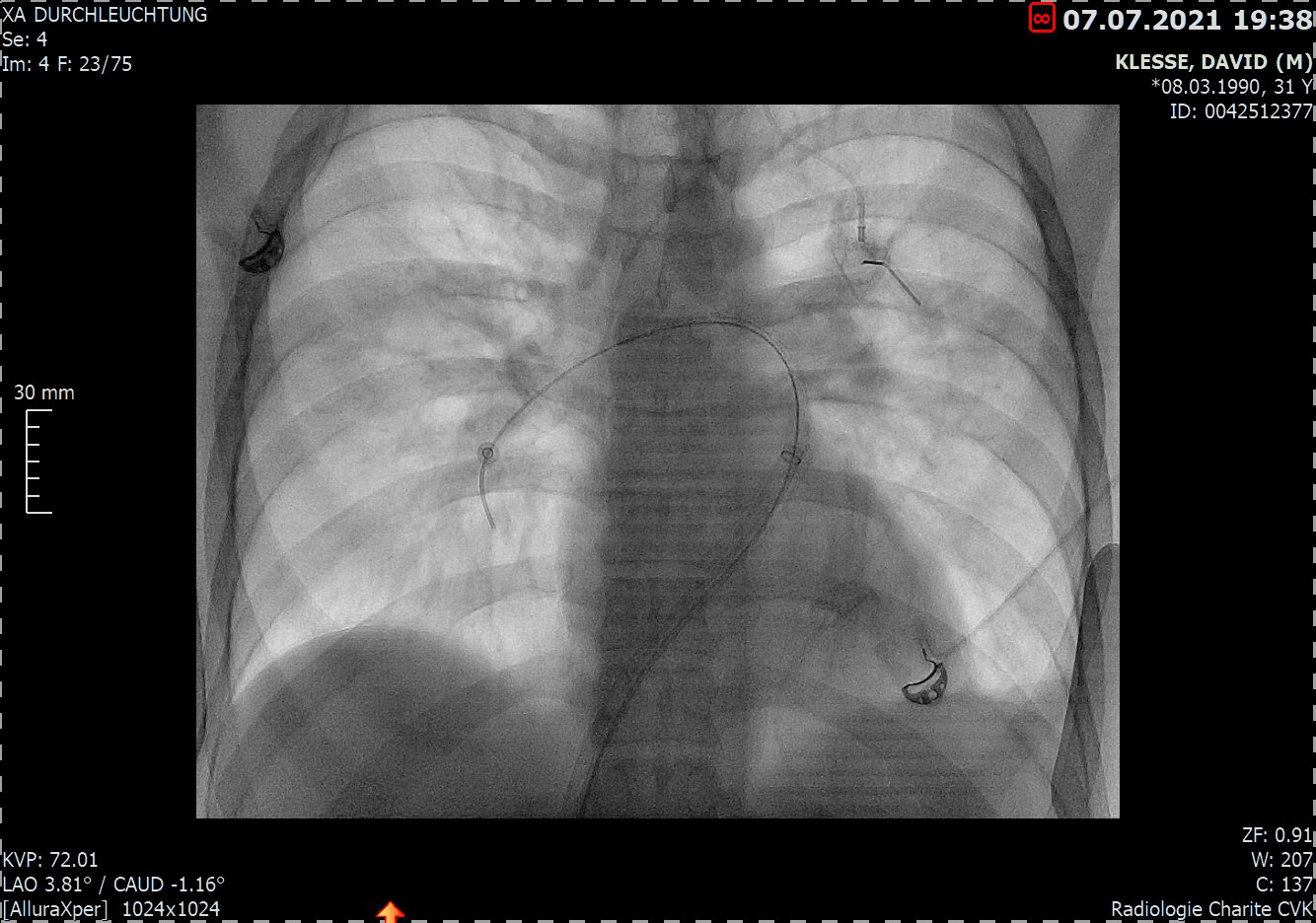

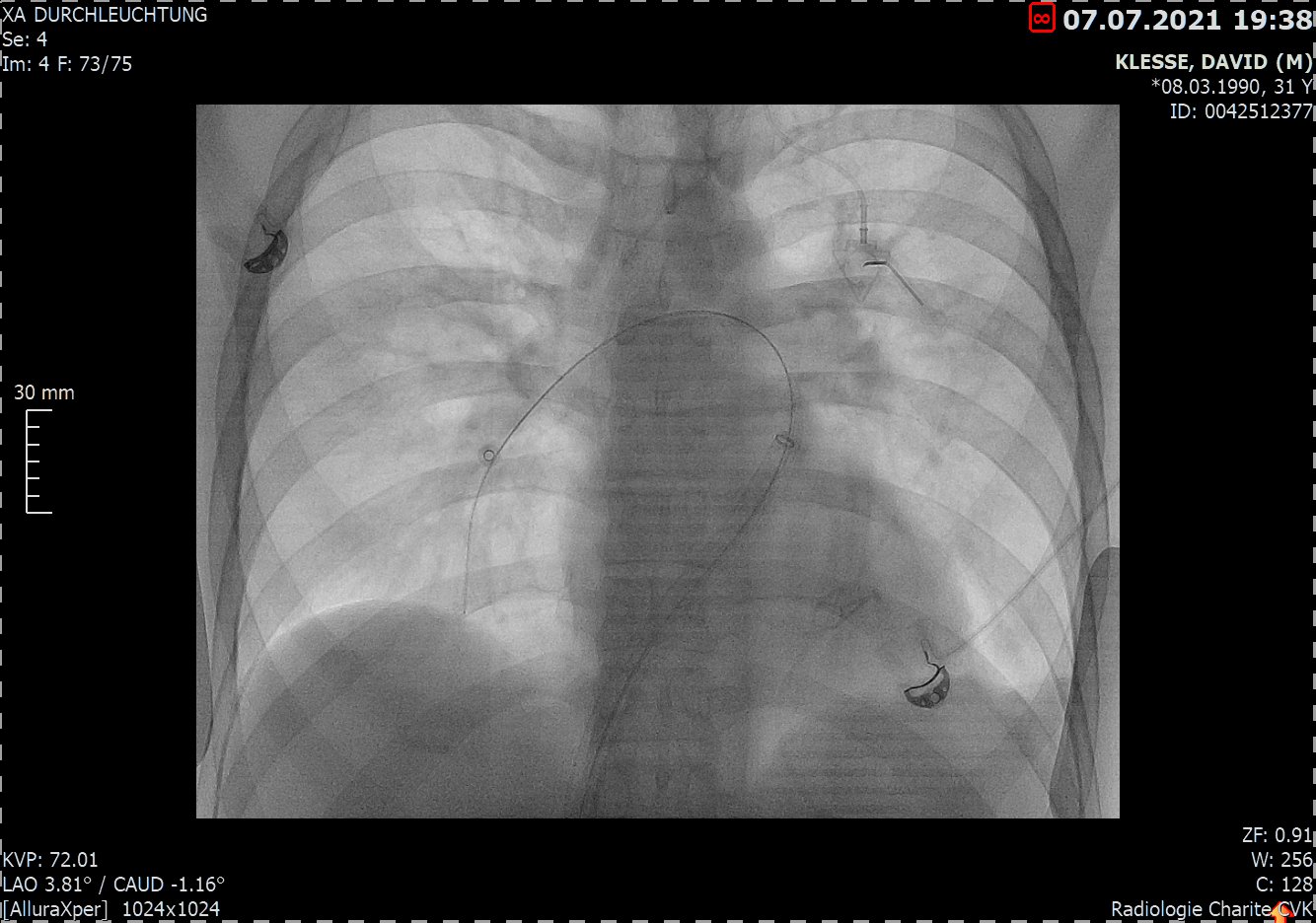

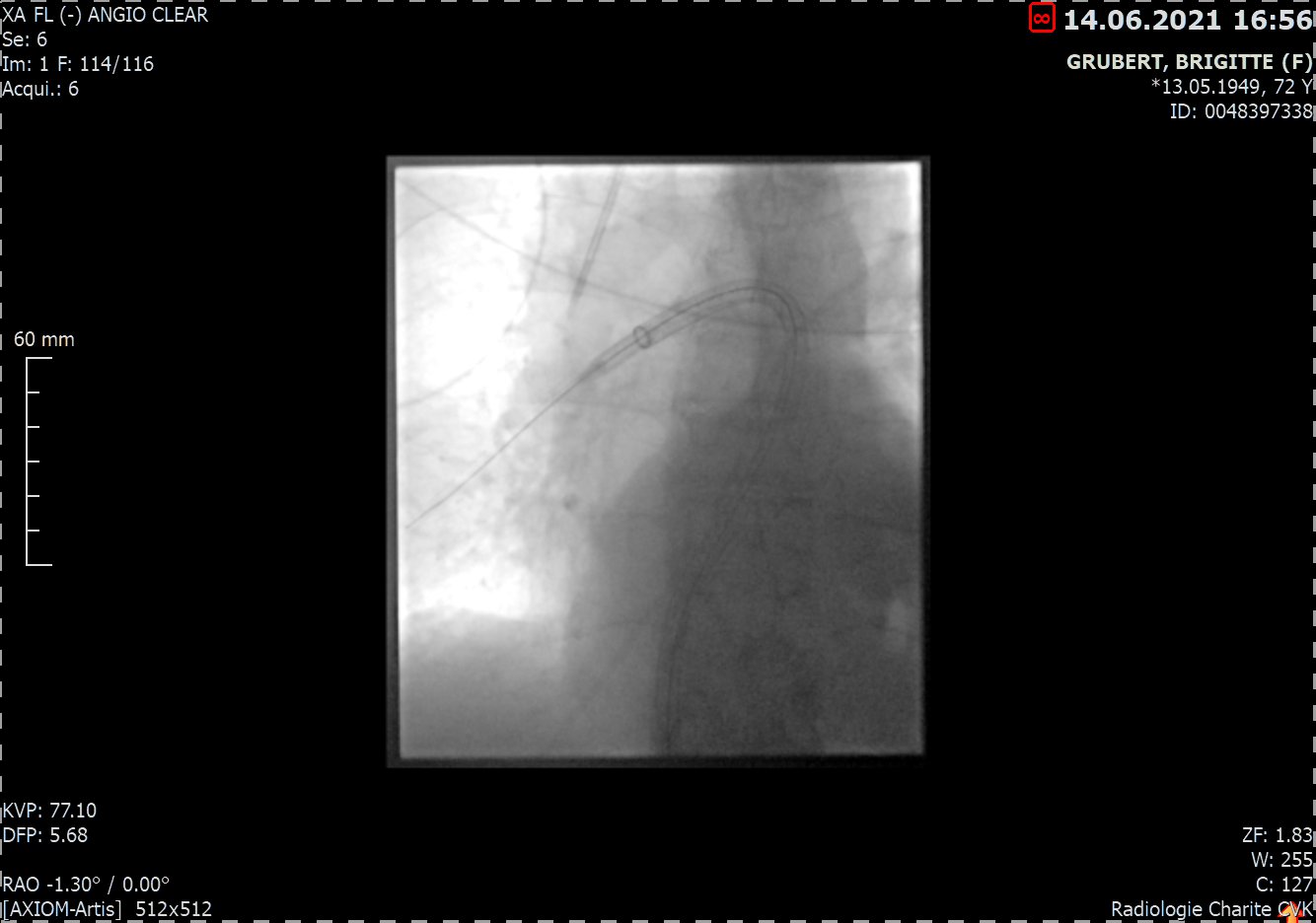

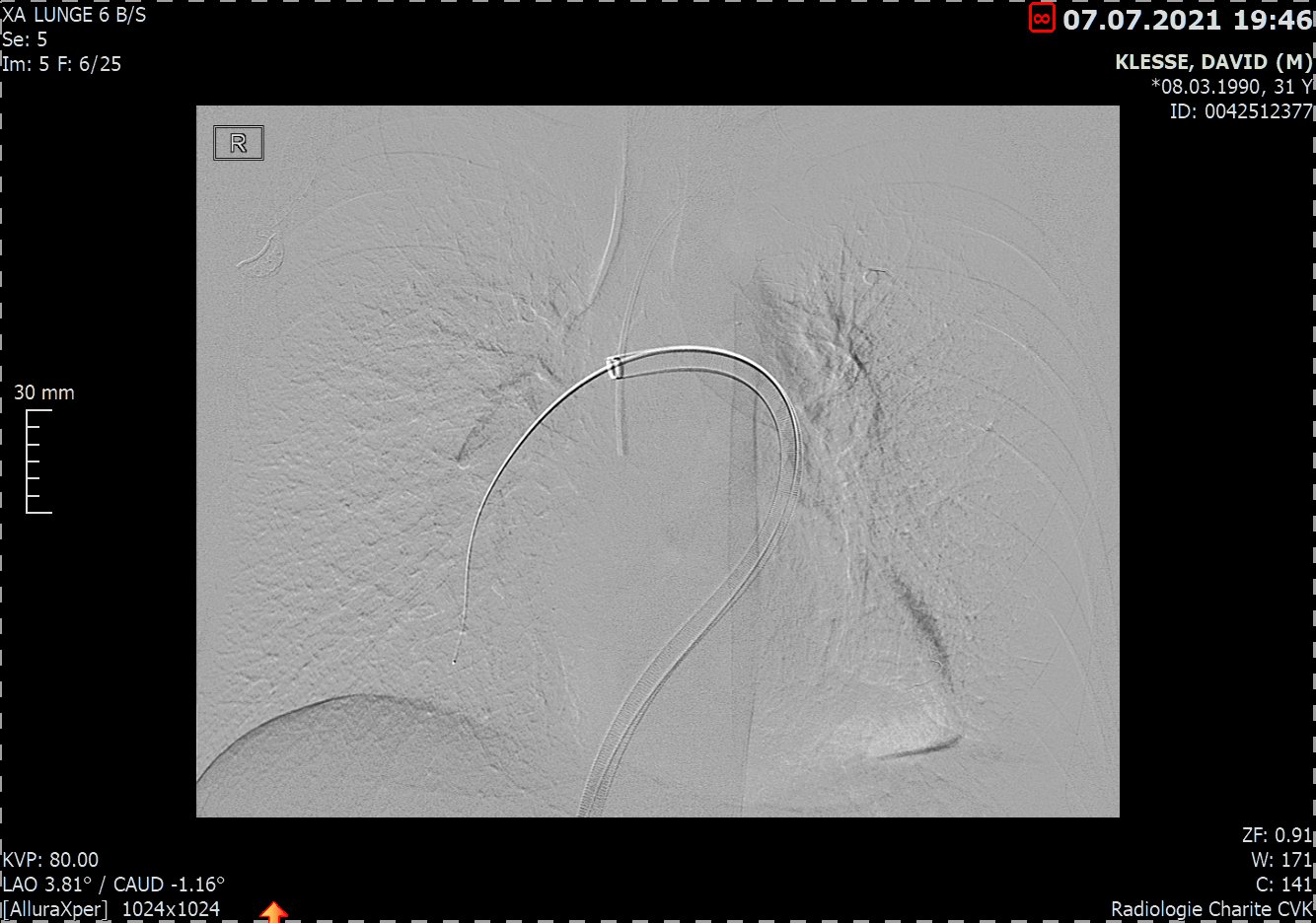

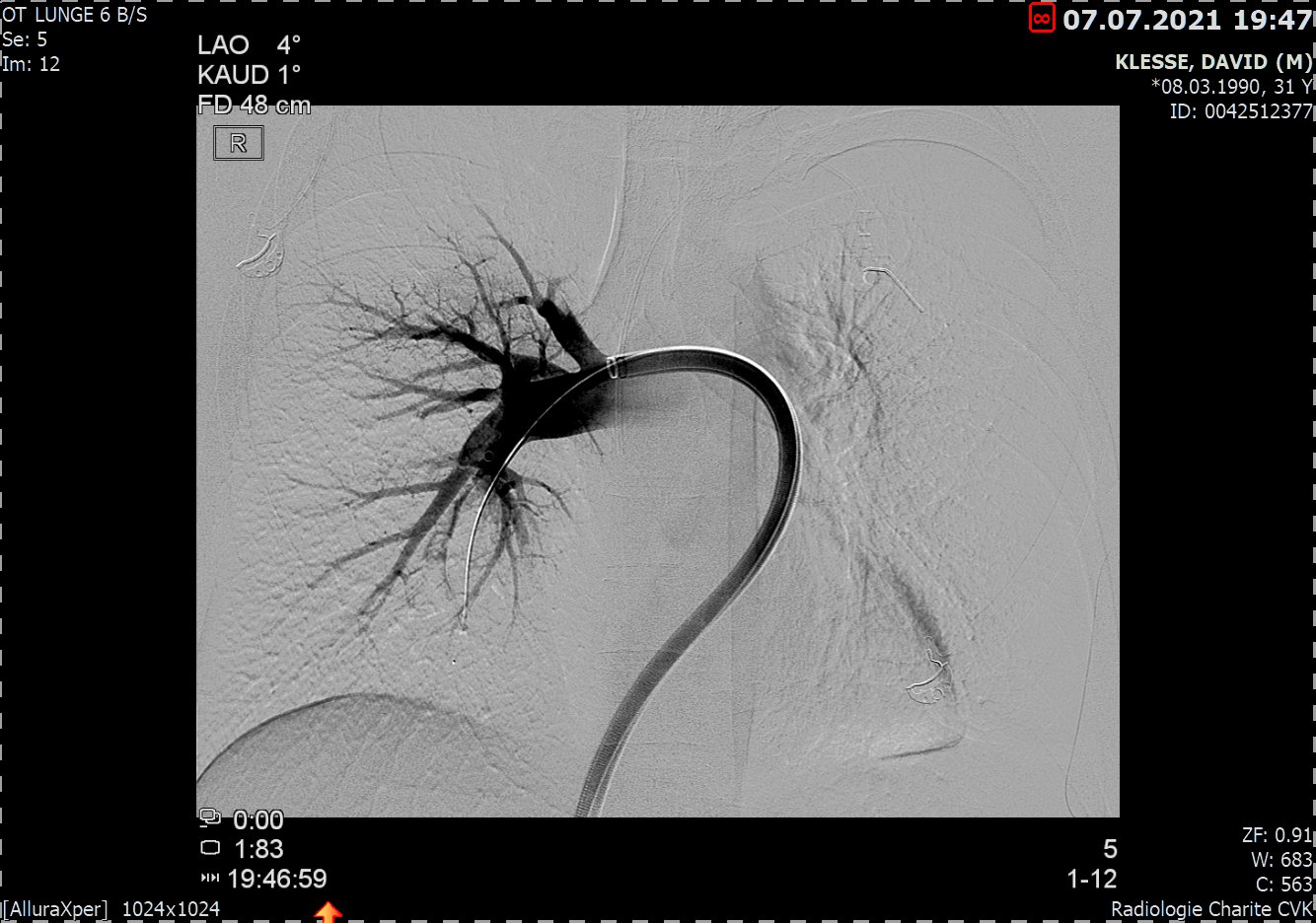


**c**

**d**

**f**

**e**

**b**

**a**

**e**

**Supplement Figure 2** Representative images from a thrombectomy procedure

In (a) a pigtail catheter is advanced into the main pulmonary artery. In (b) digital subtraction angiography (DSA) is performed to assess the thrombus burden. Pulmonary artery (PA) pressures are measured. In this particular case, the left PA was treated first which is why the 24F aspiration catheter is already located in the main pulmonary artery (c). A vertebral catheter is used to safely place an 35’’ Amplatz wire with a short floppy tip (1 cm) deep inside the right lower lobe artery (c, d). Over the wire, the FlowTriever catheter with mounted nitinol mesh disk can be inserted to disrupt clot (e, disks marked with arrows; picture taken from another case). With the inner mandrel inserted (f, picture taken from another case), the aspiration guide catheter can be advanced safely into position for thrombectomy (g). The inner mandril is removed and a vacuum is created with a 60 ml syringe and retained with a closed stopcock. Rapid release of the vacuum allows for thrombus aspiration. In (h), the cleared right PA is depicted. PA pressure measurements can be repeated.

,

**Supplement table 1** Peri-interventional effectivity measurements (data at both time points)

|  | Pre | Post | Mean diff. | Sign. |
| --- | --- | --- | --- | --- |
| PAP in mm Hg (n = 20/27) | 35.9 ± 9.6 | 26.1 ± 9.0 | 9.8 ± 12.0 | 0.002 |
| Heart rate in bpm (n = 27/27) | 109.4 ± 22.5 | 82.8 ± 13.8 | 26.6 ± 20.1 | < 0.001 |
| SAPS II score (n = 23/27) | 37.8 ± 15.3 | 31.2 ± 13.3 | 6.5 ± 13.8 | 0.020 |
| TAPSE on TTE in mm (n = 11/27) | 17.4 ± 7.3 | 16.8 ± 6.3 | 0.6 ± 11.4 | 0.873 |
| sPAP on TTE in mm Hg (n = 9/27) | 60.1 ± 17.9 | 52.7 | 7.4 ± 15.7 | 0.193 |

Values are mean ± SD.

Abbreviations:

Bpm: beats per minute; PAP: pulmonary artery pressure; SAPS II score: simplified acute physiology score II; sPAP: systolic pulmonary artery pressure; TAPSE: tricuspid annular plane systolic excursion; TTE: transthoracic echocardiography
